# Neurofibromatosis Type 1 Presenting With a Mesenteric Plexiform Neurofibroma and Epididymitis

**DOI:** 10.7759/cureus.111013

**Published:** 2026-06-17

**Authors:** Hannah Crotty, Gabriel Rodriguez-Santiago, Cynthia Reyes, Trisha Larkin, Ryan Dean

**Affiliations:** 1 Pediatrics, BayCare, Tampa, USA; 2 Pediatrics, AdventHealth Florida, Tampa, USA; 3 Pediatric Medicine, BayCare, Tampa, USA; 4 Pediatric Surgery, BayCare, Tampa, USA; 5 Hematology and Oncology, BayCare, Tampa, USA; 6 Pediatrics, University of South Florida Morsani College of Medicine, Tampa, USA

**Keywords:** abdominal mass, café-au-lait macules, epididymo-orchitis, neurofibromatosis 1 (nf1), pediatric surgery, precocious puberty (pp)

## Abstract

A 10-year-old male with a prior history of right epididymitis presented to the emergency department with a one-day history of right lower quadrant abdominal and right testicular pain. His initial examination was notable for café-au-lait macules and axillary freckling. An evaluation with renal ultrasound demonstrated mild left hydronephrosis, and subsequent testicular ultrasound was concerning for right epididymo-orchitis. A right lower quadrant abdominal ultrasound revealed a heterogeneous mass, and a CT of the abdomen and pelvis with and without contrast confirmed a large 14 cm × 16 cm × 16 cm hypoenhancing mass extending through the right lower mesentery and enveloping mesenteric vessels. An abdominal mass biopsy revealed a plexiform neurofibroma, and the patient met the criteria for neurofibromatosis type 1. He underwent resection of the plexiform neurofibroma, and further imaging revealed other neurofibromas. This case report highlights the importance of considering anatomic explanations for recurrent epididymitis in sexually naïve males and the importance of visual awareness during the physical examination, as neurocutaneous findings can influence the final diagnosis.

## Introduction

Neurofibromatosis type 1 (NF1) is a genetic neurocutaneous disorder resulting in a variety of clinical features including the development of nerve cell tumors [1,2]. While NF1 most often presents in childhood, there can be sequelae, such as the delayed growth of plexiform neurofibromas in teenage and young adult patients [1-3]. There are well-established diagnostic criteria; however, atypical presentations may delay recognition and diagnosis [1]. NF1 often involves multiple organ systems and requires multidisciplinary management from early childhood into adulthood.

Epididymitis is a common cause of scrotal pain in adults, yet is an uncommon diagnosis in pediatrics, especially before puberty and sexual debut. Adults often develop epididymitis secondary to sexually transmitted infections, bladder outlet obstruction, or urologic procedures [4]. In pre-pubertal patients, however, it is important to consider anatomical explanations for epididymitis [5].

We report the case of a 10-year-old male who presented with neurocutaneous findings of NF1 and recurrent epididymitis, which led to the diagnosis of a large plexiform neurofibroma requiring resection. This case report illustrates a unique presentation of NF1 and spotlights the importance of a thorough physical examination and investigating anatomical pathology in sexually naïve males presenting with epididymitis.

## Case presentation

A 10-year-old male with a prior history of right epididymitis presented to the emergency department with a one-day history of right lower quadrant pain and right testicular pain with swelling.

On presentation, he was febrile and tachycardic, but in no acute distress. His physical examination was significant for mild right lower quadrant tenderness without rebound or guarding, and his right testicle was erythematous with tenderness to palpation. He had Tanner stage III development; both testes were descended, and a cremasteric reflex was intact. The integumentary examination revealed axillary freckling (Figure 1) and over 10 café-au-lait macules (Figure 2).

**Figure 1:**
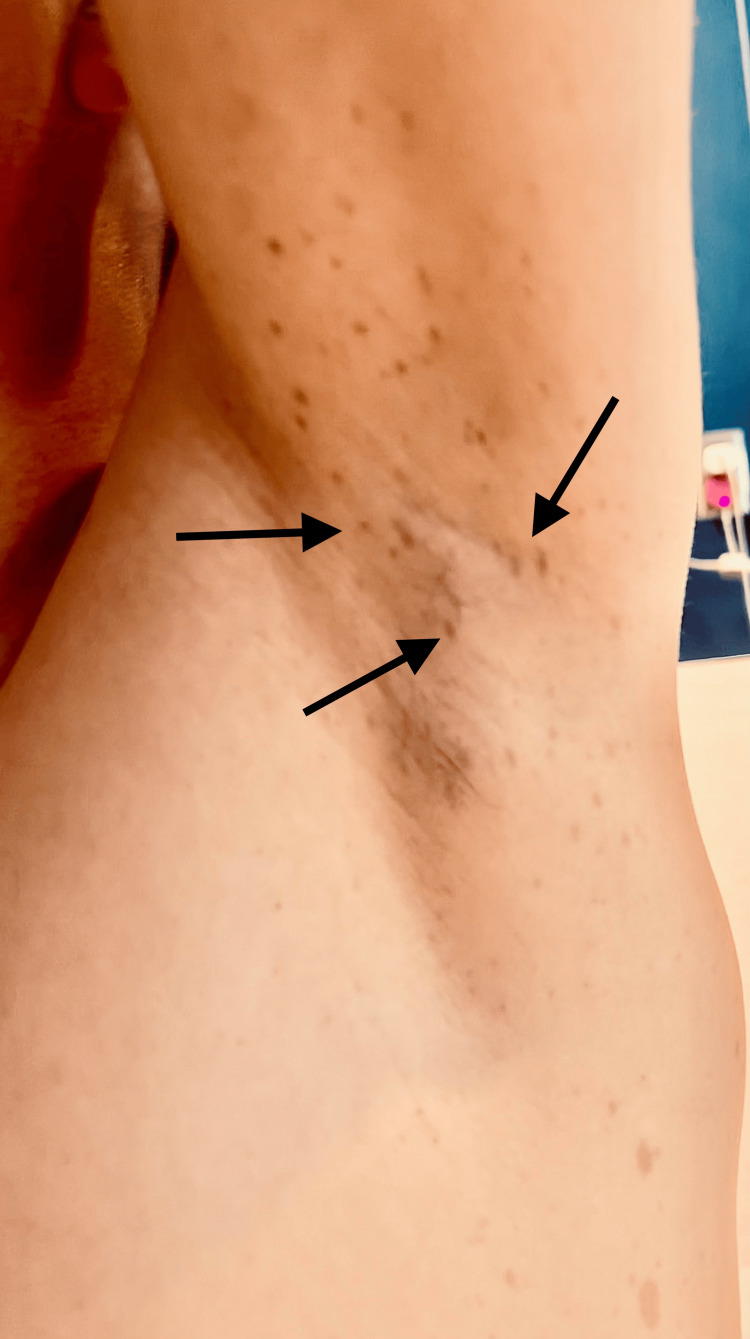
Axillary freckling on the patient’s examination.

**Figure 2:**
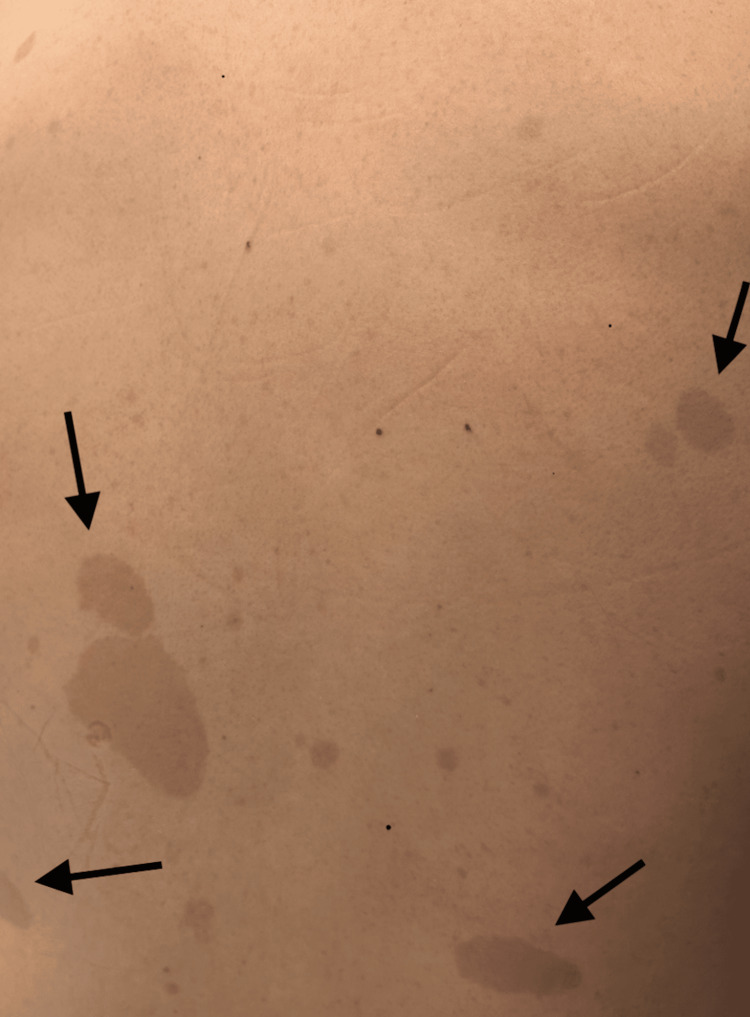
Café-au-lait macules located on the patient’s back.

Past medical history included evaluation by genetics at the age of five due to three café-au-lait macules concerning for NF1. A pediatric ophthalmology evaluation did not find evidence of optic gliomas or iris hamartomas. Based on his limited presentation, he did not meet criteria for NF1 and was lost to follow-up. The patient reported pubertal changes starting around age seven, and he had not pursued any evaluation for precocious puberty. Additionally, he was previously diagnosed with right epididymitis five months before presentation and treated with a course of antibiotics that the family could not recall. The patient had never been sexually active.

His initial workup was significant for leukocytosis to 29,300/µL and pyuria on a urinalysis with 3+ leukocyte esterase and innumerable white blood cells. A renal ultrasound showed mild left hydronephrosis, and a testicular ultrasound demonstrated right epididymo-orchitis. A right lower quadrant abdominal ultrasound revealed no evidence of appendicitis but instead identified a heterogeneous mass. Pediatric surgery and pediatric urology were consulted and recommended further imaging. A CT of the abdomen and pelvis with and without contrast revealed a large 14 cm × 16 cm × 16 cm hypoenhancing mass extending through the right lower mesentery and enveloping mesenteric vessels (Figure 3). Additional findings included urinary bladder wall enhancement, left distal ureteral urothelial thickening, and mesenteric lymph node enlargement. He was started on ceftriaxone and metronidazole for epididymo-orchitis.

**Figure 3:**
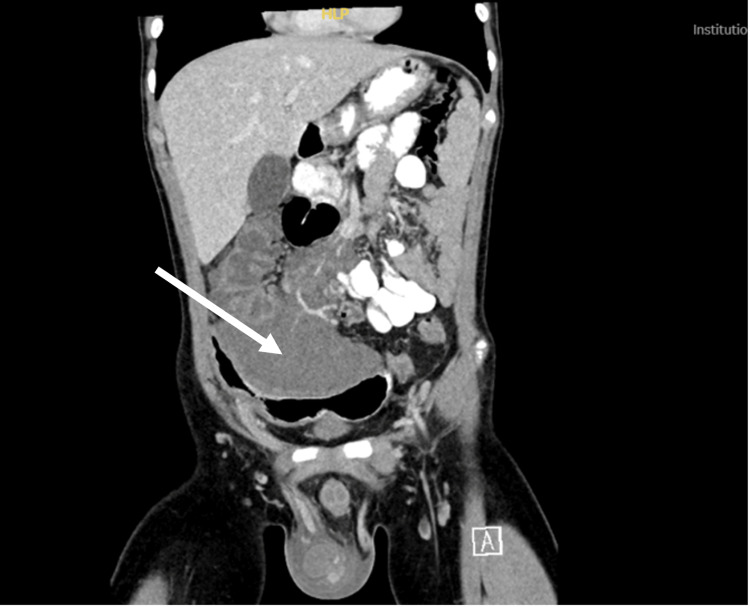
CT of the abdomen and pelvis with and without contrast demonstrating a 14 cm × 16 cm × 16 cm hypoenhancing mass extending through the right lower mesentery.

Based on the CT findings, pediatric hematology-oncology was consulted to help assess for malignancy. Serum lactate dehydrogenase and uric acid levels were within normal limits, while germ cell tumor markers were negative. Endocrine evaluation consisted of hormonal testing and a bone age scan. An X-ray of the left hand and wrist revealed a bone age of 14 years. Labs consisted of a follicle-stimulating hormone level of 5.6 mIU/mL (Tanner stage III range per lab reference for age), luteinizing hormone level of 4.67 IU/L (reference range: 0.6-12.1 IU/L), and testosterone level of 110 ng/dL (reference range: 240-871 ng/dL). Gonadotropin-releasing hormone (GnRH) stimulation test was deferred to outpatient, and the patient was referred to endocrinology after discharge. A diagnostic laparoscopy with biopsy revealed a cystic-appearing mass involving the mesentery of the distal ileum, and pathology confirmed a plexiform neurofibroma. He underwent open resection of the mesenteric neurofibroma with side-to-side ileal-ileal anastomosis due to neurofibroma adherence to the distal ileum (Figure 4). No major blood vessels were involved, though multiple mesenteric blood vessels required ligation. The procedure was tolerated well without complications. Surgical margins and four lymph nodes were negative for tumor invasion. The urine culture grew *Pseudomonas aeruginosa*, and the patient completed seven days of ciprofloxacin based on sensitivities.

**Figure 4:**
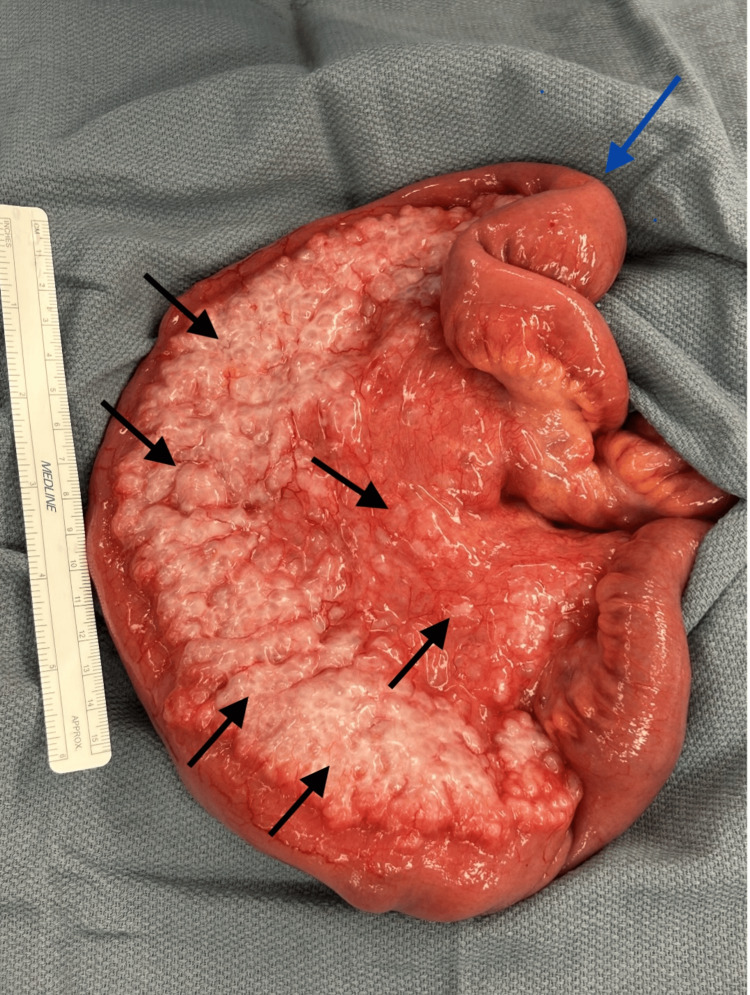
Intraoperative measurement of the mesenteric plexiform neurofibroma. The black arrows are pointing to the mesenteric tumor. The blue arrow is pointing to the ileum.

His neurocutaneous examination findings and neurofibroma supported the diagnosis of NF1. To evaluate for other lesions and possible central precocious puberty, the patient underwent an MRI of the brain and spinal cord. Imaging revealed small foci of hyperintensity on T2/fluid-attenuated inversion recovery in the globus pallidus, and a 2.4 cm × 0.7 cm × 1.0 cm enhancing lesion in the L4 vertebral body, all suspicious for neurofibromas (Figure 5). Ophthalmology evaluation did not discover any ocular manifestations of NF1.

**Figure 5:**
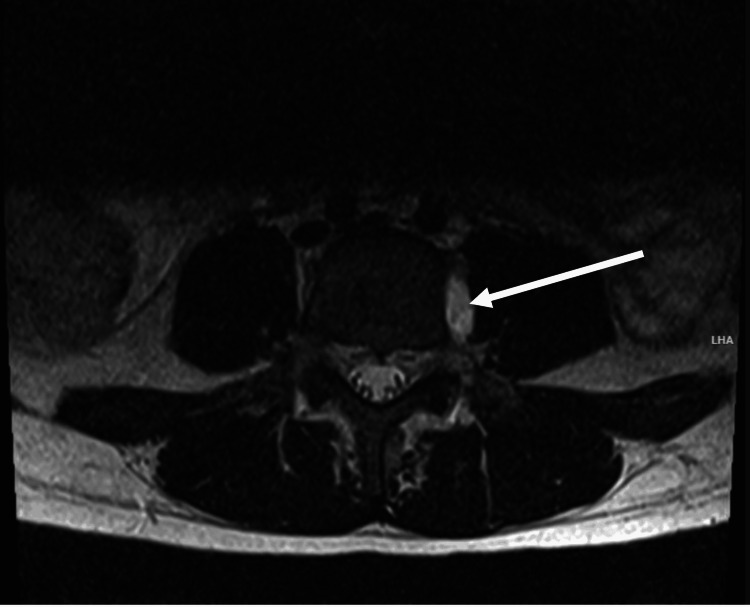
Paraspinal L4 vertebral body enhancing lesion suspicious for a neurofibroma.

After recovery from the surgical procedures and infection, the patient was discharged home pending follow-up with neuro-oncology, genetics, endocrinology, ophthalmology, infectious disease, surgery, and urology. The neuro-oncology team recommended a trial of a MEK pathway inhibitor called selumetinib, but the family declined due to concern for cardiac toxicity necessitating serial echocardiograms.

## Discussion

Neurofibromatosis is a neurocutaneous disorder divided into three subtypes, which are clinically distinct from each other. NF1 has an incidence of 1 in every 2,500 live births and accounts for 96% of cases of neurocutaneous disorders [1,2]. NF1 has an autosomal dominant inheritance pattern with loss of function of the *NF1* gene and the development of tumors along nerve cells. To meet the diagnostic criteria, a patient must have at least two of the following features: six or more café-au-lait macules; axillary or inguinal freckling; two or more iris Lisch nodules; two or more neurofibromas or one plexiform neurofibroma; distinctive osseous lesions such as sphenoid dysplasia; optic gliomas; a parent with NF1; or findings of a pathogenic *NF1* gene variant [1,2]. Overall, 97% of individuals with NF1 will meet the above clinical criteria by age 8 and 100% by the age of 20 [3]. Of note, the diagnostic criteria of NF1 changed in 2021, adding pseudoarthrosis of a long bone and a pathogenic *NF1* gene variant to the list, as well as clarifying that café-au-lait macules and axillary freckling must be bilateral [6]. NF1 remains a clinical diagnosis based on these features; thus, genetic testing is not required to make the diagnosis. As an example, our patient met the diagnostic criteria for NF1 based on clinical findings alone without the need for further genetic testing. He had more than six café-au-lait macules, bilateral axillary freckling, and subsequent evaluation revealed multiple neurofibromas including a plexiform neurofibroma. An additional unique feature of this case was the patient’s precocious puberty. According to Cheuiche et al., precocious puberty in boys is classified as the development of secondary sexual characteristics before the age of nine [7]. Our patient reported pubertal changes started around the age of seven but could not specify particular changes. [7]. As mentioned previously, the patient had evidence of puberty during our examination consistent with Tanner stage III pubic hair distribution as well as testicular enlargement [7]. Furthermore, hormonal testing during his hospitalization was consistent with pubertal development corresponding to a Tanner stage III male. Central precocious puberty can occur in children with NF1 if there are optic pathway gliomas involving the hypothalamic and sellar regions [8,9]. Our patient’s imaging did not reveal optic pathway involvement or neurofibromas affecting the hypothalamic-pituitary axis. While precocious puberty has been reported in children with NF1 in the absence of optic pathway gliomas, the underlying mechanism remains poorly understood [9]. Consequently, the patient’s precocious puberty remains in question. A GnRH stimulation test was recommended to be completed by endocrinology as an outpatient, but he has not followed up yet.

Aside from his examination features and eventual diagnosis of NF1, this patient initially presented with recurrent epididymitis. Epididymitis is one of the most common causes of scrotal pain in men aged 18 to 50. However, in pediatrics, the incidence of epididymitis is rare and typically occurs in post-pubertal adolescents. Pre-pubertal causes are typically associated with anatomical anomalies such as vesicoureteral reflux causing *Escherichia coli* infections, or by hematogenous spread during systemic bacterial infections [5]. Meanwhile, post-pubertal causes are more associated with sexually transmitted infections such as *Chlamydia trachomatis* or *Neisseria gonorrhoeae*. Enteric organisms can also cause epididymitis in adults in the setting of bladder outlet obstruction or urologic procedures [4]. Given the recurrence of epididymitis in our peri-pubertal sexually naïve patient, further workup including imaging for anatomic anomalies was prudent and helped determine the diagnosis of NF1. While we cannot definitively conclude this patient’s abdominal neurofibroma directly caused venolymphatic obstruction leading to recurrent epididymitis, imaging and intraoperative findings demonstrated the abdominal mass enveloping the distal ileum and the mesenteric vessels. It is possible the mass may have been further impeding the retroperitoneal testicular veins as well. Furthermore, there may have been some degree of urinary obstruction given contralateral hydronephrosis, left distal ureteral urothelial thickening, and urinary bladder wall enhancement on imaging. A voiding cystourethrogram would have provided additional insight into the presence of a functional or intermittent obstruction of the urinary system and is a limitation of this case report. Since resection of his mesenteric plexiform neurofibroma, the patient followed up with hematology/oncology six months later and at that time did not report a recurrence of epididymitis. This case report emphasizes the importance of keeping a broad differential in a sexually naïve male presenting with recurrent epididymitis. Upon a review of the literature, we did not find a case report describing epididymitis associated with a mesenteric plexiform neurofibroma.

Plexiform neurofibromas are benign peripheral nerve sheath tumors characterized by diffuse, infiltrative growth and occur in approximately 20% of children with NF1 [10]. These tumors may arise in virtually any location and can be associated with overlying skin changes, including hyperpigmentation, hypertrichosis, or a characteristic “bag of worms” texture [11]. Plexiform neurofibromas are thought to be congenital lesions with highly variable growth rates [12]. Periods of accelerated growth have been reported during adolescence and pregnancy, suggesting hormonal influences [13,14]. Supporting this hypothesis, Cunha et al. demonstrated growth hormone receptor immunopositivity in the majority of plexiform neurofibromas examined in a small cohort [13]. It is therefore plausible that our patient’s mesenteric plexiform neurofibroma had been present since birth and underwent accelerated growth closer to the time of presentation. Importantly, plexiform neurofibromas carry an increased risk of malignant transformation into malignant peripheral nerve sheath tumors, which are associated with significant morbidity and mortality [14].

Management of plexiform neurofibromas is highly individualized and depends on tumor size, location, growth behavior, and involvement of adjacent structures. Complete surgical resection is often challenging due to the infiltrative nature of these tumors, frequently necessitating a multidisciplinary approach [15]. Until recently, treatment options for unresectable plexiform neurofibromas in children were limited. Targeted inhibition of the MEK pathway has led to the development of selumetinib, an oral selective MEK inhibitor and the first FDA-approved medical therapy for children with NF1-associated unresectable plexiform neurofibromas. Clinical trials have demonstrated partial tumor reduction in approximately 70% of treated patients, along with improvements in pain and functional outcomes [16,17]. Although selumetinib was recommended for our patient, the family elected to decline medical therapy.

## Conclusions

NF1 is a neurocutaneous disorder that can impact multiple organ systems with a lifetime of potential complications. Examination findings of café-au-lait macules and axillary freckling should raise clinical suspicion for further investigation. In addition, recurrent epididymitis is rare in sexually naïve males and warrants further workup for anatomic explanations. This patient’s unusual presentation led to further imaging and the patient’s diagnosis of NF1 with a large abdominal plexiform neurofibroma requiring resection. Mesenteric plexiform neurofibromas can grow considerably large before they are detected; sometimes this is not until the neurofibroma compresses nearby structures, leading to pain and complications. Surgical resection can be difficult; fortunately, a new oral MEK inhibitor, selumetinib, has shown promise in reducing tumor size.
